# Cyclodextrins as Active Therapeutic Agents: Beyond Their Role as Excipients

**DOI:** 10.3390/ph18101592

**Published:** 2025-10-21

**Authors:** Andreea Silvia Pirvu, Renata-Maria Varut, Diana-Maria Trasca, George Alin Stoica, Kristina Radivojevic, Sirbulet Carmen, Cristian Cosmin Arsenie, Cristina Popescu

**Affiliations:** 1 Department of Biochemistry, University of Medicine and Pharmacy of Craiova, 200349 Craiova, Romania; andreea.pirvu@umfcv.ro (A.S.P.); kristinaradivojevic03@gmail.com (K.R.); 2 Research Methodology Department, Faculty of Pharmacy, University of Medicine and Pharmacy of Craiova, 200349 Craiova, Romania; renata.varut@umfcv.ro; 3 Department of Internal Medicine, University of Medicine and Pharmacy of Craiova, 200349 Craiova, Romania; 4 Department of Pediatric Surgery, Faculty of Medicine, University of Medicine and Pharmacy of Craiova, 200349 Craiova, Romania; 5 Department of Anatomy, University of Medicine and Pharmacy, Discipline of Anatomy, 200349 Craiova, Romania; carmen.sirbulet@umfcv.ro (S.C.); arsenie_cristian@yahoo.com (C.C.A.);

**Keywords:** cyclodextrins, inclusion complexation, cholesterol extraction, lipid raft remodeling, gene therapy, siRNA/mRNA delivery, lysosomal cholesterol mobilization, vaccine adjuvant, detoxification

## Abstract

Cyclodextrins (CDs) have traditionally been recognized as excipients that enhance solubility and stability of drugs. However, growing evidence shows that CDs themselves can act as active therapeutic agents. Their unique supramolecular properties enable them to interact with biological membranes, mobilize cholesterol, and modulate immune responses. This review highlights four therapeutic areas where CDs demonstrate particular promise. First, in gene and mRNA therapy, cationic CD derivatives form nanoparticles that protect nucleic acids, promote endosomal escape, and achieve targeted delivery. Second, in neurodegenerative disorders such as Niemann–Pick type C and Alzheimer’s disease, hydroxypropyl-β-CD facilitates cholesterol clearance and reduces pathological lipid accumulation. Third, in detoxification, the γ-CD derivative sugammadex exemplifies a clinically approved agent that encapsulates neuromuscular blockers to reverse anesthesia. Finally, CDs have emerged as safe vaccine adjuvants, inducing robust systemic and mucosal immunity with reduced IgE responses compared to alum. Together, these examples illustrate a paradigm shift: CDs are not only versatile excipients but also active molecules with direct therapeutic effects. Future translation will require careful optimization of safety, scalability, and regulatory compliance, but CDs are poised to contribute meaningfully to next-generation medicines.

## 1. Introduction

Cyclodextrins were first isolated in the late 19th century, but it was not until the latter half of the 20th century that their potential in pharmaceutical formulations was fully recognized [1]. Over the ensuing decades, these cyclic oligosaccharides have transitioned from being viewed solely as solubilizing agents and stabilizers to being acknowledged as compounds with intrinsic biological activity. Initially, cyclodextrins attracted attention in pharmaceutical science because they were able to improve the aqueous solubility and stability of poorly soluble drugs. These effects are not intrinsic properties of cyclodextrins themselves but rather the direct result of their ability to form noncovalent host–guest inclusion complexes with a wide variety of drug molecules [2,3,4,5]. This ability is largely attributed to their distinctive toroidal structure, which presents a hydrophilic exterior and a hydrophobic cavity, thereby allowing them to encapsulate lipophilic compounds in a reversible manner. As our understanding of cyclodextrin chemistry deepened, researchers began to uncover that these host–guest interactions are not merely passive processes. Rather, they can actively mediate biological responses at both cellular and systemic levels. For example, recent studies have demonstrated that cyclodextrins are able to modulate the composition of lipid rafts within cell membranes—a process that can influence receptor clustering and ultimately trigger specific intracellular signaling pathways [6,7]. This discovery has broadened the scope of cyclodextrin applications beyond drug solubilization to include direct therapeutic interventions. In parallel, significant progress has been made in the chemical modification of cyclodextrins. A recent editorial by Kfoury et al. (2025) highlighted the revival of cyclodextrins as active pharmaceutical ingredients, with examples in cholesterol modulation, rare diseases, and viral infections [8]. While that article provided a concise perspective, the present review builds upon this foundation by offering a comprehensive and systematically structured synthesis of cyclodextrins’ therapeutic roles, including gene and mRNA delivery, vaccine adjuvants, microbiome modulation, oncology applications, and detailed safety and toxicity considerations [8]. Techniques such as methylation, hydroxypropylation, and sulfobutylether substitution have been employed to generate derivatives with markedly enhanced aqueous solubility, reduced toxicity, and improved pharmacological profiles [9,10,11]. These modifications not only optimize the inclusion complex formation but also allow for a more targeted interaction with biological membranes and receptors. As a result, cyclodextrin derivatives are now being explored as active agents in diverse therapeutic applications, including gene therapy—where they serve as carriers for nucleic acids—and as modulators of immune responses, thereby offering new strategies for vaccine development and immunotherapy [12,13]. The discovery of such properties has prompted renewed interest in the broader therapeutic potential of CDs in various fields, including gene therapy, neurodegenerative diseases, detoxification, and immunomodulation.

## 2. Cyclodextrins: Physicochemical Properties and Biological Mechanisms

### 2.1. Physicochemical Properties

**Figure 1:**
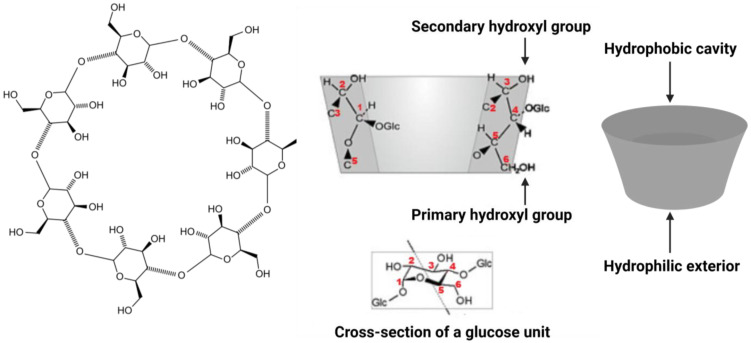
Schematic representation of a cyclodextrin molecule (β-CD) highlighting its cyclic structure and toroidal (truncated cone) shape. A cross-section (center) shows how glucopyranose units are arranged, with secondary hydroxyl groups at the wider rim (top) and primary hydroxyls at the narrow rim (bottom). This orientation creates a hydrophilic exterior (surface rich in –OH groups) and a relatively hydrophobic inner cavity (gray) capable of hosting guest molecules.

Cyclodextrins (CDs) are cyclic oligosaccharides composed of 6, 7, or 8 D-glucopyranose units linked by α-1,4-glycosidic bonds (forming α-, β-, and γ-cyclodextrin, respectively). The glucose units are arranged in a ring, which gives these molecules a distinctive truncated cone shape (Figure 1). All the CD variants share a similar toroidal geometry: the wider rim of the torus is lined by secondary hydroxyl (–OH) groups, while the narrower rim carries the primary –OH groups. This arrangement means that the exterior of the cyclodextrin is hydrophilic (due to the many exposed –OH groups), whereas the interior of the ring forms a relatively hydrophobic cavity devoid of free –OH groups. In essence, a CD molecule presents a polar “shell” to the aqueous environment and a nonpolar “pocket” on the inside [14,15,16]. This unique architecture underlies the ability of CDs to interact with hydrophobic molecules in water. Notably, CDs are non-reducing (no free anomeric carbon) and are generally regarded as non-toxic, biocompatible molecules at typical usage levels. Their enzymatic origin (production from starch by cycloglycosyltransferases) and benign properties have made them popular in pharmaceutical, food, and biochemical applications. The three native cyclodextrins differ slightly in size and solubility due to their different ring compositions. Table 1 summarizes some key properties. The diameter of the central cavity increases from roughly ~5 Å in α-CD to ~8 Å in γ-CD, accommodating progressively larger guest molecules. All cyclodextrins are fairly water- soluble, but β-CD exhibits a markedly lower aqueous solubility (~18 g/L at 25 °C) than α- or γ-CD (Table 1). This anomalously low solubility of β-CD is attributed to intra-molecular hydrogen bonding among its seven glucose units (especially a “belt” of hydrogen bonds on the secondary –OH rim) that makes the β-CD crystal lattice more stable and harder to hydrate. In contrast, α- and γ-CD have less rigid H-bond networks, allowing them to dissolve more readily (over 100–200 g/L in water). All CDs are toroidal in shape with a height of about 7–8 Å, reflecting the roughly parallel alignment of the glucose units along the ring axis. These dimensions mean that the internal cavity of a CD is just large enough to host small organic molecules, while excluding water to some extent [17,18,19,20].

Each CD molecule has numerous hydrogen-bond donors and acceptors (e.g., 21 –OH groups in β-CD) and thus a very low octanol–water partition coefficient (Log P~–14 for β-CD). This reflects the extremely hydrophilic character of the CD exterior. Yet, the inner cavity is comparably apolar, being lined by ether-like glycosidic oxygens and hydrogen atoms (from C–H groups) that project into the cavity. In water, the CD cavity tends to contain energetically “unhappy” water molecules that are not as fully hydrogen-bonded as bulk water. This dual nature—a hydrophilic outer surface and a hydrophobic inner cavity—is the defining physicochemical feature of cyclodextrins and is central to their biological mechanisms [21,22,23].

### 2.2. Inclusion Complex Formation

One of the most important properties of cyclodextrins is their ability to form inclusion complexes (host–guest complexes) with other molecules. The relatively hydrophobic cavity of a CD can encapsulate a guest molecule (or a portion of it), isolating that guest from the bulk aqueous environment. No covalent bonds are formed or broken in this process—the guest is held within the CD’s cavity by non-covalent forces. The driving forces for complex formation are primarily van der Waals interactions and the hydrophobic effect (the tendency of nonpolar surfaces to avoid contact with water). The interior of the cyclodextrin provides a snug, oil-like microenvironment into which suitably sized hydrophobic molecules can insert. This expulsion of water from the cavity upon guest binding is thermodynamically favorable: water molecules confined in the CD cavity are relatively energetically unstable (they cannot form a full complement of hydrogen bonds inside the apolar cavity) and are sometimes called “high-energy water”. When a guest molecule displaces these cavity waters, the water molecules are released into bulk solvent where they can form more stable hydrogen bonds. The net result is an enthalpic gain—effectively, the CD and guest gain stability by releasing strained water molecules and by maximizing favorable van der Waals contacts in the host–guest complex. This mechanism explains why inclusion complexation in CDs is often an enthalpy-driven process (in contrast to the entropy-driven hydrophobic aggregation seen in other contexts) [24,25,26,27,28]. Several additional factors influence CD complex formation: the size/shape complementarity between host and guest is critical (guests that are too large or too small fit poorly and bind weakly). For example, β-CD’s cavity is “just right” for many aromatic and aliphatic compounds, which is one reason β-CD is so widely used in inclusion studies. Specific polar interactions can also play a role: while the interior of the cavity is mostly nonpolar, the rims of the CD have hydroxyl groups that can form hydrogen bonds or dipole interactions with functional groups on the guest as it enters or sits in the cavity. Such interactions can orient the guest within the CD or modestly improve binding affinity, but they are usually secondary to the predominant hydrophobic interactions. The inclusion complex is typically in rapid equilibrium with free guest and free cyclodextrin in solution, with stability constants on the order of 10^2^–10^5^ M^−1^ for many drug–CD complexes. In other words, the binding is often reversible and moderate in strength, allowing the guest to be released under the right conditions (e.g., dilution, displacement by a competitor, or changes in environment) [29,30,31,32].

## 3. Engineering and Design of Cyclodextrin Derivatives

### 3.1. Monosubstitution of CDs

When native cyclodextrins are not sufficient, chemists modify them to gain new properties. Early attempts created single-substituted versions, but making pure products was difficult because each molecule has several possible sites for modification [33]. More recently, significant interest has arisen in grafting cyclodextrins onto magnetic nanomaterials, leveraging their distinctive dimensions and physicochemical traits [34]. In one approach, β-cyclodextrin was selectively derivatized and anchored onto Fe_3_O_4_ nanoparticles, yielding magnetically responsive conjugates that simplify the enantiomeric separation process. This immobilization strategy not only facilitates magnetic recovery but also enhances the structural robustness of the cyclodextrin shell [35].

### 3.2. Two-Position Modification

Natural cyclodextrins present two distinct hydroxyl functionalities on their narrower rim, and steric crowding often influences reactivity. In practice, the secondary C 2 hydroxyl is more amenable to substitution than the C 3 position [36]. For example, Liu and colleagues synthesized an α CD derivative by tethering an oligo(ethylene glycol) linker capped with photo switchable moieties, yielding a light responsive dimer whose hydrodynamic radius shifts dramatically upon irradiation; the terminal amine also offers a convenient handle for further size modulation [37,38]. In parallel work, β CD was appended to azobenzene units, though competing side reactions under varied conditions necessitated optimized protocols to drive the desired coupling [37]. Because native CDs dissolve poorly in water, new derivatives were made. One of the most successful, hydroxypropyl-β-CD, showed much better solubility and improved absorption of steroid hormones. Other versions, like dimethyl-β-CD, were less effective, showing the importance of careful design [38]. Zhao et al. reported two regioisomeric 2 O (2 hydroxybutyl) β CDs that acted as superior chiral selectors compared to both native β CD and 2 HP β CD in separating racemic pharmaceuticals [39].

Oxidizing β-CD improved its solubility and also allowed it to bind ferrocene, creating a complex with electrical conductivity useful for biosensing [40]. Further advances include C 2 organoselenium–modified CDs characterized by circular dichroism and NMR—demonstrating enhanced enantioselectivity [41]—and novel β CD variants synthesized via copper sulfate catalysis or alternative C 2 modification routes, which were probed for host–guest affinities using hydrocinnamic and adamantane carboxylates [42,43]. Finally, a γ CD analogue bearing pyrene carbonyl groups prepared in DMF exhibited strong excimer fluorescence and formed stable inclusion complexes with 1 borneol [44].

### 3.3. Three-Position Displacement

Modification of the C-3 hydroxyl is notably more challenging than at C-2 because of its reduced acidity, and only a handful of investigations have addressed trisubstituted cyclodextrins [45,46,47]. In recent work, Miyawaki and colleagues generated both linear and hyperbranched supramolecular polymers by linking modified CD monomers; these assemblies exhibited polymer-like behavior attributable to heterotypic inclusion complexes reinforced by hydrophobic and hydrogen-bond interactions [48]. Employing a similar strategy, another group achieved a β-CD derivative in 30% isolated yield with over 90% regioselectivity, demonstrating that the cinnamyl substituent can be readily exchanged for acetyl or hydrogen groups; 2D NMR analyses confirmed the precise substitution pattern in these monoderivatized CDs [49]. Using a per-O-methylation route with tert-butyloxycarbonyl protection for the amino functionality, researchers synthesized 3-monoamino permethylated α- and β-CDs, benefiting from the carbamate’s stability under reaction conditions and its facile deprotection [50]. Masurier et al. prepared an aqueous β-CD–Cu(II) inclusion complex with some derivatives already commercialized [42], and Martina’s team applied ultrasound- and microwave-assisted techniques to produce pure altrose isomers of 3-deoxy-3-azido CDs across three cyclodextrin scaffolds with improved yields and purity [43]. Finally, Suzuki et al. achieved a tri-point modification of γ-CD with pyrene carbonyl in DMF, confirming its enhanced guest-binding capacity (Table 2 and Table 3) [44].

### 3.4. Mechanistic Basis of Cyclodextrin Biological and Therapeutic Effects

Cyclodextrins are classically considered excipients that enhance the solubility, stability, or bioavailability of active pharmaceutical ingredients. However, certain derivatives also exhibit intrinsic biological actions that can translate into therapeutic benefits. For instance, hydroxypropyl-β-cyclodextrin has been shown to mobilize cholesterol in Niemann–Pick type C disease, while sugammadex acts as an active drug by encapsulating neuromuscular blockers. Thus, while the primary role of cyclodextrins remains as pharmaceutical excipients, specific contexts and chemical modifications allow them to act as active therapeutic agents. The following mechanisms illustrate this duality.

Cyclodextrins interact with cell membranes by extracting cholesterol and other lipids from the bilayer. This depletion of membrane cholesterol can disrupt lipid rafts and alter cell signaling. In a therapeutic context, 2 hydroxypropyl β cyclodextrin has been shown to solubilize and remove cholesterol deposits in tissues, dissolving cholesterol crystals within atherosclerotic plaques. By clearing these lipid accumulations, cyclodextrin treatment reduced plaque size and inflammation in animal models, highlighting how membrane lipid extraction underlies its benefit in cholesterol-related disorders [80,81,82].

Cyclodextrins can also modulate immune responses and have been explored as vaccine adjuvants. Certain cyclodextrin derivatives (e.g., hydroxypropyl β cyclodextrin, HPβCD) can stimulate innate immunity and enhance antigen specific adaptive responses. Notably, HPβCD has an excellent safety profile and has been shown to induce immune cell activation (e.g., T helper 2 cell proliferation) when co administered with antigens. Unlike traditional aluminum-based adjuvants, cyclodextrin adjuvants elicit strong antibody production with minimal IgE release. In intranasal influenza vaccine models, HPβCD acted as an effective mucosal adjuvant, boosting IgA/IgG levels and providing enhanced protection, demonstrating cyclodextrins’ potential as safe immune response modulators in vaccines [83,84,85].

**Figure 2:**
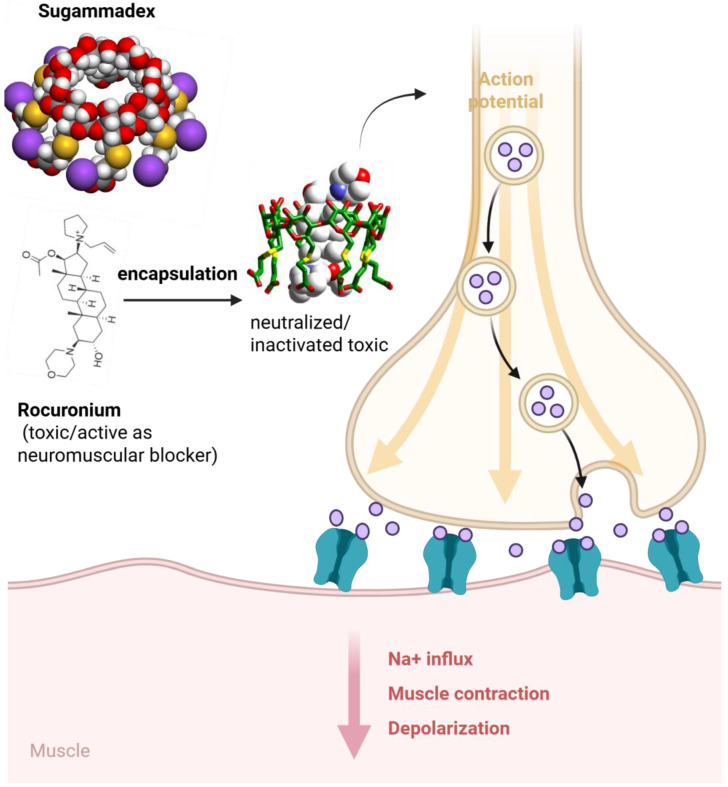
Mechanism of action of sugammadex: encapsulation of the neuromuscular blocker rocuronium within the γ-cyclodextrin cavity, leading to neutralization of its effect and restoration of neuromuscular transmission, sodium influx, and muscle contraction. https://app.biorender.com/illustrations/68de5801c2852769683f0f22 (accessed on 14 October 2025).

Cyclodextrins inclusion abilities enable them to sequester toxic substances or drugs, reducing toxicity. A prime example is sugammadex, a modified γ cyclodextrin designed to encapsulate the neuromuscular blocker rocuronium. By forming a tight inclusion complex, sugammadex rapidly binds and inactivates the muscle relaxant, reversing neuromuscular blockade in patients. This mechanism—essentially “soaking up” the toxin—underscores cyclodextrins’ detoxification role (Figure 2).

Researchers have also created cyclodextrin hybrids for heavy metal detox; for instance, a β cyclodextrin–deferasirox conjugate can simultaneously chelate iron (via the deferasirox moiety) and encapsulate cholesterol. Such multifunctional cyclodextrins illustrate how toxin or metal sequestration (through inclusion or coordination) can be leveraged to decrease toxic ion levels and protect tissues [86,87].

Cyclodextrins are valuable building blocks in targeted drug delivery systems and controlled release formulations. By attaching cyclodextrins to polymers, nanoparticles, or hydrogels, drugs can be directed to specific sites and released in a controlled, stimuli responsive manner. For example, pH sensitive cyclodextrin based hydrogels have been developed that swell and release their drug payload preferentially in acidic environments (such as tumor tissue or the stomach). In one design, a cationic β cyclodextrin hydrogel showed pH dependent swelling and drug release, demonstrating how a cyclodextrin formulation can achieve targeted, controlled drug delivery. More broadly, cyclodextrin containing nanocarriers (including “nanosponges” and cyclodextrin–polymer conjugates) have been shown to prolong drug release, improve tissue targeting (e.g., tumor uptake), and reduce off target toxicity. These properties make cyclodextrin based delivery systems powerful tools for achieving localized and sustained therapeutic action [88,89,90,91,92].

Therefore, cyclodextrins can act both as classical excipients and, in selected cases, as active therapeutic agents, a paradigm shift supported by preclinical and clinical evidence.

## 4. Safety Profile and Toxicity

Native β-cyclodextrin (β-CD) is known to have limited aqueous solubility, which can lead to accumulation in tissues at high doses. This accumulation has been associated with potential nephrotoxicity, as the compound may deposit in renal tissues and interfere with normal kidney function. In contrast, derivatized forms such as hydroxypropyl-β-cyclodextrin (HP-β-CD) have been chemically modified to improve solubility and reduce toxicity. HP-β-CD, for instance, demonstrates a markedly enhanced safety profile, making it a more suitable candidate for therapeutic applications. Toxicological studies indicate that cyclodextrins are generally well tolerated when administered within established therapeutic dose ranges [93,94]. Nonetheless, the potential for adverse effects increases with chronic administration, particularly at high parenteral doses, where renal tubular vacuolization and hepatic alterations have been reported in preclinical studies [95,96]. Long-term use of cyclodextrin-based formulations may therefore require careful monitoring of renal and hepatic functions, as these organs play key roles in metabolism and excretion [97,98]. The risk of cumulative toxicity, particularly in vulnerable patient populations, necessitates a cautious approach when considering prolonged treatment regimens. The reversible binding of drugs to cyclodextrins contributes not only to their beneficial controlled-release profiles but also to their safety characteristics. The ability to form inclusion complexes can protect active molecules from enzymatic degradation, yet it also underscores the importance of understanding the release kinetics in relation to potential toxic effects. For example, excessive retention of a drug within its cyclodextrin complex may reduce the availability of the free drug needed for therapeutic action, while also posing a risk of localized toxicity if the complex accumulates in sensitive tissues [99,100,101,102,103,104].

## 5. Methods

**Figure 3:**
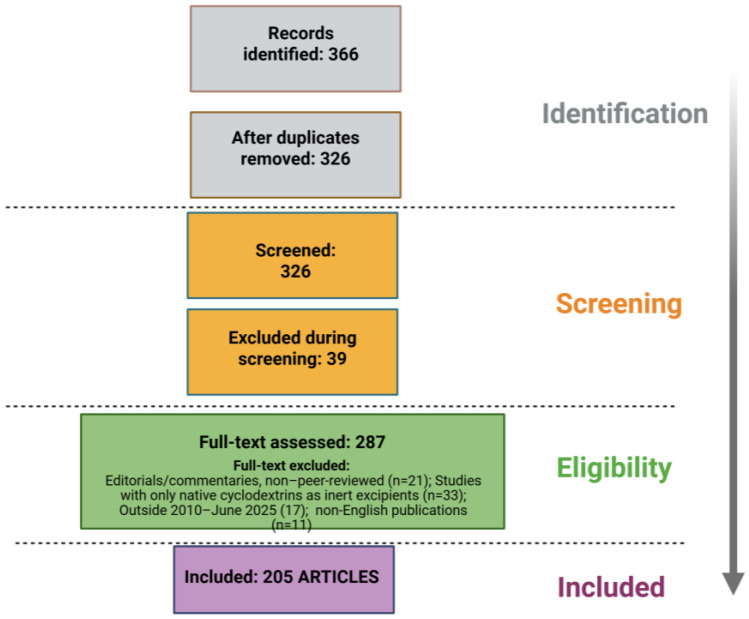
PRISMA Flow Diagram of Study Selection Process.

This narrative review integrates elements of a systematic search guided by core PRISMA recommendations. We searched PubMed, Google Scholar, and Web of Science for English-language, full-text articles published between January 2010 and June 2025 using Boolean combinations of “cyclodextrin” with “derivative,” “modified,” “functionalized,” “polymer,” “supramolecular,” “nanoparticle,” and “stimuli-responsive.” To ensure comprehensive coverage, we also hand-screened the bibliographies of key reviews and primary studies. Eligible reports were required to focus on the chemical engineering of cyclodextrin derivatives—including synthesis routes such as etherification, click chemistry, and cross-linking—and to provide physicochemical characterization (e.g., NMR, FTIR, DSC, XRD) or explore applications in drug delivery, theranostics, or targeted therapies (for conditions ranging from cystic fibrosis and Duchenne muscular dystrophy to neurodegenerative disorders and blood–brain barrier transport). We excluded conference abstracts, editorials, non-peer-reviewed commentaries, and studies confined to native cyclodextrins as inert excipients. From each included study, we extracted details on derivative type, degree of substitution, analytical methods, stimuli-responsive behavior, and in vitro or in vivo performance, recording these data in a standardized spreadsheet. Rather than a quantitative meta-analysis, our goal was to produce a thematic narrative that maps current design strategies, highlights innovative functional systems, and identifies translational research opportunities in the field of engineered cyclodextrin therapeutics (Figure 3).

## 6. Application in Therapy

### 6.1. Cyclodextrin-Based Systems for Gene and mRNA Delivery

Gene- and RNA-based therapeutics have emerged as promising strategies for a wide range of diseases, from inherited genetic disorders to cancers and infectious diseases. These approaches involve introducing nucleic acids (DNA, siRNA, or mRNA) into patients’ cells to produce a therapeutic protein or to modulate gene expression. However, naked DNA or RNA is inherently unstable in the biological environment: nucleases rapidly degrade unprotected nucleic acids, and large anionic molecules poorly traverse the hydrophobic plasma membrane. Without a suitable delivery vehicle, most of the administered genetic material never reaches its intracellular target. Conventional systems also have drawbacks: viral vectors, while efficient at transduction, can trigger strong immune responses and limit repeat dosing, whereas non-viral carriers such as cationic lipids or polymers may be cytotoxic or inflammatory. This has spurred interest in developing alternative non-viral systems that balance stability, efficacy, and safety—among them are cyclodextrin-based carriers.

**Figure 4:**
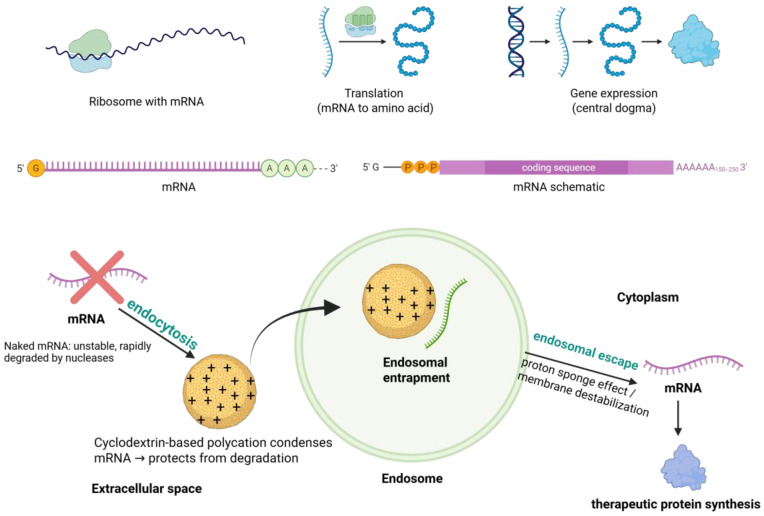
Mechanism of cyclodextrin-based mRNA delivery: formation of CD–mRNA complexes, cellular uptake, endosomal entrapment, and subsequent endosomal escape enabling translation into therapeutic proteins. https://app.biorender.com/illustrations/68de1c7065f09faf01d710d2 (accessed on 14 October 2025).

Over the past decade, cyclodextrins have been engineered into diverse nucleic acid delivery systems. Their mechanisms of action include:

- Encapsulation and Protection from Nucleases. Cyclodextrins can encapsulate or complex nucleic acids to shield them from enzymatic degradation. CD-based polycations form polyplexes with DNA, siRNA, or mRNA, prolonging nucleic acid half-life and ensuring that a meaningful fraction of the dose reaches target cells [105,106,107,108].
- Enhanced Cellular Uptake and Endosomal Escape. When conjugated with cationic molecules, CDs condense nucleic acids into nanoparticles that cells can internalize via endocytosis. CDs may also transiently modulate membranes to facilitate uptake. Once inside endosomes, CD-based carriers can promote cytosolic release through mechanisms such as the proton sponge effect or pH-sensitive linkages, preventing degradation in endo-lysosomal compartments [109,110,111].
- Chemically Modified CDs and Polyrotaxane Architectures. Native CDs have limited affinity for large nucleic acids, but cationic modifications or polyrotaxane structures enhance binding and condensation. These supramolecular architectures increase complex stability while enabling controlled disassembly and release [112,113,114,115].
- Stimuli-Responsive and Targeted Delivery. A crucial advantage of cyclodextrin carriers is their favorable safety profile. CDs are derived from starch, and many derivatives (e.g., hydroxypropyl-β-CD) are already FDA-approved excipients with low toxicity and immunogenicity. Incorporating CDs into cationic polymers can mitigate polymer-induced cytotoxicity, and biodegradable CD-based polymers degrade into non-toxic sugars. This supports repeat administration, an essential requirement for chronic gene therapy or mRNA vaccination [116,117,118]. CD-based systems can be designed with pH- or enzyme-sensitive linkages that trigger release in the acidic endosome or protease-rich tumor microenvironment. Ligands such as folate, transferrin, or antibodies can be attached for cell-specific targeting, improving therapeutic precision (Figure 4) [119,120].

Applications in mRNA delivery. Cyclodextrin polycations can spontaneously form nanoparticles with mRNA through gentle self-assembly, yielding polyplexes that protect mRNA against ribonucleases and rapid clearance. These systems can be further stabilized with hydrophilic polymers (e.g., PEG) and functionalized with targeting ligands. Smart designs introduce pH-sensitive or enzyme-responsive linkages, enabling stimuli-triggered release of the mRNA in the cytosol [121,122]. Similarly to RNA, DNA binding by cationic cyclodextrins occurs primarily through electrostatic interactions between the positively charged groups introduced onto the cyclodextrin scaffold and the negatively charged phosphate backbone of DNA. This interaction leads to the condensation of plasmid or oligonucleotide DNA into nanoscale polyplexes, which provide protection against enzymatic degradation and facilitate cellular uptake. Chemical modifications such as quaternary ammonium or polyethyleneimine grafting further enhance complex stability, allowing the formation of nanoparticles with suitable size and surface charge for transfection. Moreover, supramolecular architectures including polyrotaxanes or amphiphilic cyclodextrin assemblies offer multivalent binding sites and dynamic adaptability, thereby improving both condensation and controlled release of DNA cargo. Importantly, cyclodextrin-based DNA carriers have been investigated in diverse applications ranging from plasmid DNA vaccines to gene replacement strategies and have demonstrated promising biocompatibility and reduced cytotoxicity compared to traditional cationic polymers [123].

Preclinical studies highlight the therapeutic promise of CD-based mRNA systems. In vaccine development, CD–polyethyleneimine conjugates have enhanced antigen expression and immune responses in vivo. In cancer immunotherapy, CD-mRNA nanoparticles encoding tumor antigens or cytokines have induced local protein expression and slowed tumor growth in animal models. For monogenic diseases such as cystic fibrosis or Duchenne muscular dystrophy, inhalable or injectable CD-based carriers are being tested to deliver functional proteins repeatedly, overcoming size and immunogenicity limitations of viral vectors [124,125,126,127,128,129].

### 6.2. Cyclodextrin-Based Delivery of siRNA

In addition to their growing role in mRNA therapeutics, CDs have also been extensively studied as non-viral carriers for small interfering RNA (siRNA). Modified CDs, often bearing cationic or amphiphilic groups, can electrostatically complex with siRNA to form nanoscale polyplexes that protect the oligonucleotides against nuclease degradation and promote cellular uptake [124,125]. A landmark example of the translational potential of this approach is CALAA-01, a β-cyclodextrin-based nanoparticle functionalized with transferrin, which represented the first targeted siRNA delivery system tested in humans and demonstrated gene silencing in a phase I clinical trial [126].

Beyond transferrin-targeted systems, folate-appended cyclodextrins have shown enhanced delivery efficiency to tumor cells overexpressing folate receptors, thereby improving selectivity and reducing off-target effects [125]. Other derivatives, such as hydroxypropyl-β-cyclodextrin incorporated into cationic polymers, have yielded stable and biocompatible complexes capable of mediating efficient siRNA knockdown in vitro and in vivo [127]. Applications extend beyond oncology, as cyclodextrin-based nanoparticles have also been engineered for siRNA delivery in neurodegenerative disorders, including Huntington’s disease, where β-cyclodextrin derivatives facilitated neuronal uptake and silencing of mutant huntingtin expression in cellular and animal models [128].

Collectively, these studies underscore that CDs provide a versatile and relatively safe platform for siRNA therapeutics, complementing their role in mRNA delivery and expanding their potential utility across both cancer and central nervous system disorders.

### 6.3. Clinical Relevance and Translational Outlook

Cyclodextrin-enabled gene delivery systems are moving beyond the laboratory, with several examples in clinical or translational development. Cyclodextrin-based carriers have already made historic progress in oncology. The first-in-human clinical trial of a polymeric nucleic acid nanoparticle (known as CALAA-01) utilized a cyclodextrin-containing polymer to deliver siRNA to patients with solid tumors. This system included a β-cyclodextrin polycation as the core of the delivery vehicle, along with a tumor-targeting ligand and polyethylene glycol for stability. In Phase I testing, the CD-based nanoparticle showed it could successfully deliver the siRNA into tumor cells in human patients with an acceptable safety profile, demonstrating gene silencing in tumor biopsies. Although that particular therapy was an siRNA (gene silencing) approach, the same delivery principles apply to mRNA. Despite the encouraging first-in-human results of CALAA-01, including evidence of siRNA delivery and gene silencing, further development stalled due to several translational barriers. Manufacturing of cyclodextrin–polymer complexes at clinical scale proved challenging, as supramolecular assemblies introduced batch-to-batch variability and required stringent GMP standardization. Regulatory agencies also highlighted the limited toxicological data for long-term exposure to cationic polymers, as well as uncertainties regarding immunogenicity of targeting ligands. Clinically, efficacy signals were modest in early oncology trials, and patient recruitment remained limited. In parallel, the rapid advancement of lipid nanoparticles provided a competing platform that was easier to scale, better characterized, and quickly validated during the COVID-19 mRNA vaccine rollout. These factors collectively explain why early cyclodextrin-based siRNA carriers such as CALAA-01 did not progress beyond Phase I, while also underscoring the need for simpler, biodegradable, and regulatory-compliant CD derivatives in future clinical translation [129,130].

The clinical feasibility of cyclodextrin carriers has sparked further research into using them for cancer vaccines or for delivering therapeutic mRNAs (for example, mRNA encoding tumor suppressors or chimeric antigen receptors) in cancer patients. Ongoing translational studies are evaluating CD-polymer vehicles for improved targeting of metastatic tumors and minimizing systemic toxicity, with the aim of moving these into clinical trials in the near future [129,130,131].

### 6.4. Cystic Fibrosis (CF)

CF is a prime candidate for mRNA therapy because it is caused by a single gene defect (in the CFTR chloride channel) and affects accessible organs like the lungs. However, any CF treatment will require repeated delivery of the CFTR gene or mRNA to lung epithelial cells, given the cells’ turnover and the chronic nature of the disease. Cyclodextrin-based delivery systems, being non-immunogenic and suitable for inhalation formulations, are well suited for this task. Inhalable powders or aerosols containing cyclodextrin–mRNA complexes are being explored to overcome the barriers of mucus and reach the lung cells. Recently, the first clinical trials of inhaled mRNA for CF have begun (using advanced non-viral nanoparticles), underscoring the translational push in this field. Cyclodextrins can play a role by enhancing the stability and dispersibility of these inhaled formulations and by reducing inflammatory responses in the airways. For example, a hypothetical cyclodextrin-modified nanoparticle carrying CFTR mRNA could be given repeatedly via nebulizer without significant loss of efficacy to immune reactions. As these trials progress, researchers will be watching for improvements in lung function and patient outcomes, with the hope that cyclodextrin-facilitated delivery will offer a viable therapeutic option for CF patients who currently have limited treatment options beyond symptom management [132,133].

### 6.5. Duchenne Muscular Dystrophy (DMD)

DMD presents a delivery challenge due to the enormous size of the dystrophin gene/mRNA and the need for body-wide muscle transduction. Viral vectors like AAV have size limitations and patients can develop immunity to the vector, precluding redosing. Cyclodextrin-based carriers, in contrast, could potentially carry large mRNA transcripts (including full-length dystrophin, which is far larger than what AAV can package) and be administered multiple times. Although still in preclinical stages, researchers are investigating CD-containing nanoparticles for muscle delivery. For instance, cationic CD polymers could be complexed with dystrophin mRNA or with CRISPR/Cas9 mRNA and guide RNA and then injected into the bloodstream or muscle tissue. Initial experiments suggest that these nanoparticles can infiltrate muscle fibers and express functional protein, at least locally. A major clinical advantage of a successful CD-based system for DMD would be the ability to dose periodically throughout a patient’s childhood and adolescence to maintain dystrophin levels as muscles grow, something not feasible with one-time gene therapy approaches. While no cyclodextrin-mediated DMD therapy is in trials yet, the concept is being developed in translational research, and it represents a hopeful direction for a disease that remains incurable [134,135,136].

### 6.6. Infectious Diseases (Vaccines)

The recent success of mRNA vaccines for COVID-19 has highlighted the power of mRNA delivery, but current vaccines rely on lipid nanoparticles which require ultra-cold storage and can cause reactogenic side effects in some recipients. Cyclodextrin-based delivery systems are being examined as an alternative platform for next-generation vaccines against infectious diseases such as influenza, HIV, or novel emerging pathogens. CDs could improve the thermal stability of mRNA formulations (for example, forming a dry powder vaccine that remains stable at ambient temperatures) and reduce side effects by avoiding some of the inflammatory components of traditional lipid formulations. Moreover, cyclodextrins have been used as adjuvants and solubilizers in other vaccine contexts, suggesting they could simultaneously serve to modulate immune response. While no CD-based mRNA vaccine has yet reached late-stage clinical trials, early studies in animals (for example, a CD nanoparticle delivering an mRNA encoding a viral antigen) have shown effective immune protection. The translational relevance here is high: such vaccines could be easier to distribute globally and allow for rapid updates (simply by swapping the mRNA sequence) without having to reformulate entirely new delivery vehicles each time. As the field of gene therapy and vaccines moves forward, cyclodextrin-enabled delivery is likely to become an integral part of the discussion, offering a blend of safety, efficacy, and logistical advantages (Table 4) [137,138,139,140,141].

### 6.7. Cyclodextrins and Lipid Homeostasis

**Figure 5:**
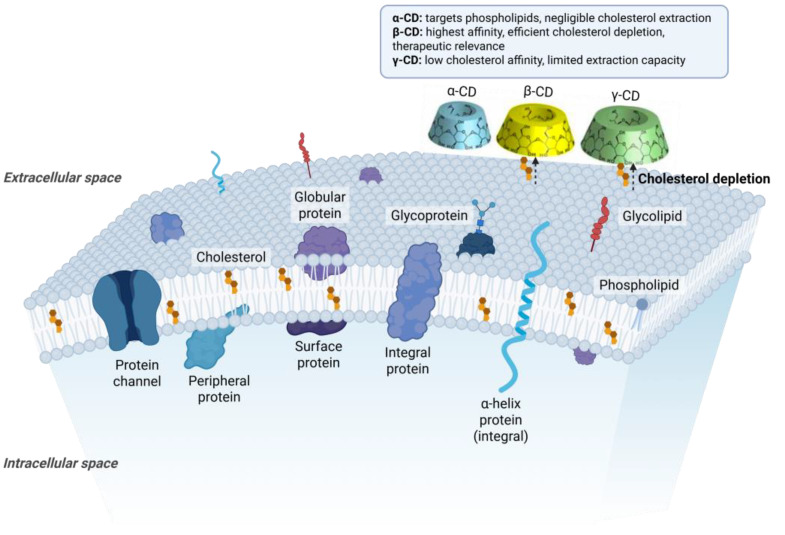
Schematic representation of cholesterol extraction by cyclodextrins (α-, β-, and γ-CD) from the plasma membrane. Cyclodextrins interact with cholesterol molecules embedded within the phospholipid bilayer, leading to cholesterol depletion and disruption of lipid rafts. This mechanism underlies their therapeutic effects in cholesterol-associated disorders such as Niemann–Pick type C disease, atherosclerosis, and Alzheimer’s disease. https://app.biorender.com/illustrations/68de0b80223dbc7b585982b7 (accessed on 13 October 2025).

Among the most studied properties of cyclodextrins in the CNS is their capacity to modulate cholesterol and lipid metabolism. Certain derivatives, especially 2-hydroxypropyl-β-cyclodextrin (HP-β-CD) and methyl-β-cyclodextrin (MβCD), can extract excess cholesterol from cell membranes and lysosomes, thereby restoring normal lipid homeostasis. This is particularly relevant in diseases like Alzheimer’s, where cholesterol accumulation within neuronal membranes has been implicated in promoting amyloidogenic processing of amyloid precursor protein. In transgenic mouse models of Alzheimer’s, administration of HP-β-CD has been shown to reduce amyloid plaque deposition and improve cognitive performance (Table 5). These effects are attributed to enhanced cholesterol efflux, normalization of membrane fluidity, and activation of lysosomal clearance pathways. In Parkinson’s disease models, cyclodextrins have similarly demonstrated the ability to reduce α-synuclein aggregation by altering lipid raft composition and promoting autophagic clearance. In Huntington’s disease, where mutant huntingtin protein aggregates and perturbs synaptic and mitochondrial function, cyclodextrins help restore cholesterol balance and protect against excitotoxicity. Methylated β-cyclodextrins have been shown in cellular models to decrease aggregation of huntingtin fragments, reduce cellular toxicity, and improve survival (Figure 5) [146,147,148,149].

### 6.8. Cyclodextrins in Neurodegenerative Disease Therapy: Roles as Delivery Enhancers and Therapeutic Facilitators

Although cyclodextrins do not exert intrinsic pharmacological effects in the classical sense, certain derivatives have demonstrated therapeutic relevance in neurodegenerative disorders. Their primary contribution lies in facilitating drug delivery by improving solubility, stability, and bioavailability of co-administered agents. In addition, in specific contexts such as Niemann–Pick type C disease, HPβCD has been shown to directly mobilize cholesterol and reduce lysosomal storage, thereby producing clinically meaningful outcomes. In Alzheimer’s, Parkinson’s, and Huntington’s diseases, cyclodextrins act mainly as delivery enhancers that improve the efficacy of neuroprotective or disease-modifying drugs, while also modulating lipid-related pathways that indirectly support neuronal function [152,153].

#### 6.8.1. Exploring Intranasal Administration as a Brain-Targeting Route for HPbCD

Intranasal delivery has gained attention as a less invasive alternative to intrathecal injection for accessing the central nervous system, effectively bypassing the blood–brain barrier. Although small molecules (e.g., sedatives, anxiolytics, corticosteroids) are readily administered via this route [154], high-molecular-weight agents require individual evaluation. In mice, comparative pharmacokinetic studies revealed that HPβCD reaches all brain regions following either intranasal or intrathecal administration, but peak CNS concentrations were consistently lower with the nasal approach [155].

The neuroprotective potential of intranasally delivered HPβCD was further assessed in a rat model of Alzheimer’s disease. Here, β-amyloid was stereotactically injected into the hippocampus, and HPβCD was supplied as mucoadhesive microspheres in the nasal cavity. Untreated animals exhibited elevated markers of lipid peroxidation, reactive oxygen species, and apoptosis in the hippocampus, whereas HPβCD-treated rats showed marked reductions in oxidative stress indicators. However, since the microsphere excipients (sodium alginate or chitosan) may themselves contribute to neuroprotection, the direct effects of HPβCD in this context warrant additional investigation [156].

#### 6.8.2. Niemann–Pick Disease

Hydroxypropyl-β-cyclodextrin (HPβCD) received orphan-drug designation for compassionate use in Niemann–Pick Disease type C (NPD-C) after promising animal data and the absence of other therapies; the U.S. Food and Drug Administration granted this status in 2010 and the European Medicines Agency followed suit in 2013 [157]. Early clinical investigations delivered HPβCD directly into the cerebrospinal fluid of infants under 18 months, demonstrating a rebalancing of neuronal cholesterol levels and attenuation of central nervous system lesions [150]. In a small cohort of three toddlers (30–36 months old), intrathecal HPβCD administration correlated with measurable gains in cognitive performance, swallowing reflexes, and postural control—though these observations lacked a placebo comparator [151]. A broader open-label study spanning three years, with participants ranging from 4 to 23 years, similarly reported a deceleration in disease progression [152].

By contrast, a randomized, double-blind trial involving 56 subjects (38 receiving 900 mg biweekly HPβCD intrathecally for 52 weeks versus 18 undergoing a sham procedure) found no significant difference in primary endpoints between treatment and control groups [153,154]. Alarmingly, a notable proportion of treated individuals experienced hearing impairment, echoing preclinical findings of HPβCD–induced ototoxicity in cochlear hair cells [155]. These outcomes ultimately led the FDA to withhold full approval of HPβCD for NPD-C [156], a decision likely influenced by the study’s limited sample size and the 2:1 randomization scheme [157]. Ongoing and planned trials aim to refine the clinical profile and support eventual licensure of HPβCD (commercially known as Adrabetadex) in this indication [158].

#### 6.8.3. Alzheimer’s Disease

In treated animals, HPβCD administration led to a pronounced reduction in cholesterol accumulation in the hippocampus compared with controls. Transmission electron microscopy of the corpus callosum revealed more myelinated fibers and thicker myelin sheaths, while behavioral tests indicated improvements in learning and memory, though motor coordination was unaffected. In vitro experiments using APOE4 homozygous oligodendrocytes further showed that HPβCD reduced intracellular neutral lipid deposits (cholesterol and triacylglycerides), supporting a lipid-clearing effect [159]. Since the compound shows limited permeability across the blood–brain barrier, its action is unlikely to stem from direct central effects; rather, systemic exposure suggests that peripheral mechanisms may contribute to the observed benefits [160]. This is consistent with evidence implicating peripheral APOE isoforms in Alzheimer’s disease pathogenesis [161].

In line with these observations, Yao and colleagues evaluated HPβCD in a transgenic Alzheimer’s model (Tg19959 mice expressing human APP). Mice receiving 4 mg/kg twice weekly from early postnatal stages to four months of age performed better in cognitive tasks, without changes in motor outcomes. Brain analyses showed reduced neuroinflammation and decreased β-amyloid deposition, pointing to attenuated amyloidogenic APP processing [162]. A subsequent study by the same group confirmed that reduced amyloid plaque burden was accompanied by improved spatial learning and memory in Tg19959 mice [163].

#### 6.8.4. Parkinson’s Disease

Parkinson’s disease involves the progressive loss of dopaminergic neurons in the substantia nigra, often associated with mitochondrial defects that promote toxic protein aggregates and neuronal death [164]. A major component of these inclusions is α-synuclein, a presynaptic protein whose misfolding and fragmentation impair neurotransmission [165]. Mitochondrial dysfunction has also been linked to autophagy defects, paralleling mechanisms described in Alzheimer’s models [166].

In cellular studies, H4 neuroglioma cells expressing α-synuclein–EmGFP showed a reduction in intracellular inclusions upon HPβCD treatment, mediated by enhanced autophagic clearance via LAMP-2 upregulation and increased autophagosome formation [167]. Comparable effects were observed in Parkinson’s patient–derived organoids, where HPβCD improved survival and outgrowth of dopaminergic neurons. In vivo, subcutaneous administration of HPβCD (4 g/kg twice weekly) protected mice against MPTP-induced neurotoxicity, with histology confirming preservation of midbrain dopaminergic neurons [166]. Supporting its translational potential, recent patents propose HPβCD-based strategies for safeguarding dopaminergic cells in Parkinson’s and related disorders [168,169,170].

### 6.9. Cyclodextrin-Based Drug Delivery Systems for Cancer Therapy

Conventional chemotherapeutic agents often suffer from intrinsic limitations—such as low water solubility, sensitivity to light, malodorous profiles, and a narrow therapeutic window marred by systemic toxicity—that hinder their clinical utility [171,172]. To address these challenges, current efforts are directed toward engineering advanced drug-delivery platforms capable of enhancing solubility, bioavailability, and targeted transport across physiological barriers (for example, within the tumor microenvironment, across the reticuloendothelial system, or through the blood–brain barrier) [173,174,175,176,177]. Although many new anticancer drugs have been synthesized in recent decades, their adoption has been curtailed by excessive side effects, high manufacturing costs, and poor patient adherence [174].

Cyclodextrins, owing to their unique cavity architecture and favorable safety profile, have emerged as versatile nanocarriers for encapsulating a wide range of anticancer payloads—both synthetic molecules and natural extracts—and for improving their pharmacokinetic and pharmacodynamic characteristics [171,178,179,180,181]. The following subsections will highlight recent innovations in cyclodextrin-based delivery systems that show particular promise against prevalent and high-mortality tumor types.

Bognanni and co-workers engineered cross-linked α-, β-, and γ-cyclodextrin polymers bearing either arginine–glycine–aspartic (RGD) motifs or arginine residues and loaded them with doxorubicin or oxaliplatin. These CD-based nanocarriers markedly potentiated the drugs’ antiproliferative effects in A549 and HepG2 cell models, with particularly strong enhancement of doxorubicin activity in HepG2 cells and improved oxaliplatin efficacy in both lines [182].

Wu et al. designed mesoporous silica nanoparticles capped with β-CD gates and a photo/redox-responsive azobenzene–galactose polymer, achieving efficient doxorubicin delivery and greater cytotoxicity in HepG2 cells relative to HeLa and COS7 cells [183]. In a different redox-sensitive approach, Mousazadeh and colleagues assembled folate-decorated polyethylenimine–β-CD supramolecular nanoparticles to co-deliver adamantane-conjugated doxorubicin together with hTERT-siRNA; the platform produced sustained, pH-triggered intracellular release, robust gene transfection, excellent biocompatibility/hemocompatibility, and targeted apoptosis of folate-receptor–positive cells [184].

Jia et al. reported a tumor-microenvironment-responsive nanoplatform based on an ROS-sensitive MPEG-CD-PHB polymer for combined delivery of doxorubicin and the photosensitizer purpurin-18, enabling controlled payload release and synergistic chemo-photodynamic antitumor effects [185]. Pooresmaeil and Namazi grafted β-CD onto magnetic graphene oxide to produce a tumor-targeting carrier for doxorubicin and methotrexate; this system showed preferential drug release in cancer cells versus healthy cells, suggesting improved safety and efficacy over free drug administration [186].

Vukic and co-workers formed an inclusion complex between acetylshikonin and β-CD and tested it on HCT-116 and MDA-MB-231 cells; compared with the free agent, the complex produced stronger short-term cytotoxicity (notably in HCT-116), superior long-term growth suppression in both lines, enhanced cell-cycle arrest and autophagy inhibition, and higher intracellular ROS accumulation [187]. Hu et al. complexed Saikosaponin-d with hydroxypropyl-β-CD for topical cancer applications; the inclusion converted the drug to an amorphous, more water-soluble form through hydrogen-bonding interactions and triggered apoptosis in HSC-1 cells via MAPK activation and Akt–mTOR pathway inhibition [188].

Parvathaneni’s group used sulfobutyl ether-β-CD to form an inclusion complex with afatinib, aiming to boost oral absorption by reducing P-glycoprotein efflux; complexation enhanced cytotoxicity across several cancer cell lines and improved the agent’s tumor-volume reduction capacity [189]. Xu et al. screened various CDs for solubilizing the immunomodulator NLG919 and identified hyperbranched-β-CD as the most suitable for IV formulation; this complex also potentiated paclitaxel’s cytotoxicity, consistent with suppression of IDO-1 and amplification of tumor-targeted immune responses [190].

Rodell and colleagues employed β-CD nanoparticles to deliver R848 (a TLR7/8 agonist) in multiple mouse tumor models, reprogramming the tumor immune microenvironment toward an M1 phenotype, controlling tumor growth, preventing rechallenge, and—when combined with anti-PD-1 therapy—improving immunotherapy response rates [191]. Finally, Zhang et al. synthesized a single-isomer, amphiphilic cationic CD for chlorin e6 delivery; the construct improved photodynamic therapy outcomes by offering enhanced stability, preferential cellular uptake, greater ROS generation, and increased phototoxicity [192].

#### Lung Cancer

Lung cancer remains one of the most prevalent malignancies worldwide. Despite advances that have modestly lowered its incidence, it still leads in cancer-related deaths and carries dismal survival rates across most stages. As a result, considerable research is devoted to developing more effective therapies [193,194,195,196,197]. Notably, multiple studies have investigated the incorporation of cyclodextrins into chemotherapeutic and photodynamic treatment formulations to enhance drug delivery and therapeutic efficacy (Table 6).

Other applications of CDs in cancer therapy are summarized in Table 7.

Bone malignancies represent under 1% of newly diagnosed cancers annually, yet they carry a heavy burden of morbidity and mortality [218]. A major obstacle in treating these tumors is the frequent emergence of drug resistance, which has driven efforts to create delivery platforms that concentrate therapeutics at the skeletal lesion while sparing healthy tissues. Targeted nanocarriers tailored for bone can reduce the need for high systemic doses, prolong drug residence at the tumor site, protect payloads from rapid clearance, and thereby mitigate off-target toxicity [219].

Several cyclodextrin-based strategies have shown promise in this setting. Ahmadi et al. reported a “smart” system in which a synthetic, cationic polymer coats magnetic nanoparticles that present cyclodextrin anchoring units; this construct produced pH-responsive release of methotrexate, was well tolerated by nonmalignant cells, and exhibited enhanced uptake and antiproliferative effects against Saos-2 osteosarcoma cells compared with free drug [220]. Khelghati and co-workers engineered a pH-sensitive, magnetic hyperbranched β-CD nanocarrier for doxorubicin delivery; the platform proved biocompatible, released drug preferentially under acidic conditions, and demonstrated greater cytotoxicity toward Saos-2 cells after incubation than the unformulated agent [221]. Plesselova et al. described a modular approach using polyethyleneimine as a scaffold to attach bisphosphonate targeting ligands and to host cyclodextrins as drug carriers; this multicomponent system effectively delivered doxorubicin to bone tumor cells and metastatic sites and can be readily adapted by swapping ligand or cargo modules for different therapeutic goals [222].

## 7. Conclusions

In summary, cyclodextrins represent a paradigm shift in pharmaceutical science. Their capacity to act as both excipients and active therapeutic agents underscores their versatility and potential in modern medicine. This review has highlighted the diverse therapeutic properties of CDs, including applications in gene therapy, neurodegenerative diseases, detoxification, vaccine development, and microbiome modulation. While significant challenges remain in translating these findings into clinical practice, the accumulated preclinical and early clinical evidence is promising. Continued research and collaboration across disciplines will be critical to harnessing the full potential of cyclodextrins, ultimately leading to safer and more effective therapeutic interventions for a wide array of diseases.

## Figures and Tables

**Table 1:** Native Cyclodextrin Types and Key Properties (at 25 °C).

Cyclodextrin	Glucose Units in Ring	Cavity Diameter (Å)	Water Solubility (g/L)
α-CD	6	4.7–5.3	~145
β-CD	7	6.0–6.5	~18
γ-CD	8	7.5–8.3	~232

**Table 2:** Cyclodextrin derivatives and synthetic strategies employed for the preparation of chromatographic stationary phases, with corresponding analytical applications in chiral separation and molecular recognition.

Derivative and Modification	Preparation Method	Analytical Application	Ref.
α-CD grafted via PEG-sol–gel	Polyethylene glycol–based sol–gel	Stationary phase for aromatic isomer separation	[51]
Mono-6-deoxy-benzimide-β-CD	Direct amide coupling	Enantioseparation of rigid analytes	[52]
Cationic β-CDs (4 variants)	Quaternization; coated on silica	CSP tested with various alcohol eluents	[53]
Cationic β-CDs (4 variants; pyCDCl best)	Quaternization	Enantiomer resolution	[54]
Mono-azido-β-CD “clicked” onto silica	Cu-catalyzed azide–alkyne cycloaddition	Stable CSP with high enantioseparation	[55]
R-configured β-CD derivatives	Stereospecific substitution	CSP; molecular docking to probe chiral recognition	[56]
Vinylene-functionalized cationic β-CD on vinylized silica	Vinyl click-chemistry	Novel CSP	[57]
Chiral monolithic phases from novel CD derivatives	In situ polymerization on capillary	Monolithic CSP for multiple chiral compounds	[58]

**Table 3:** Cyclodextrin derivatives and their chemical modification strategies, highlighting structural innovations, key functional properties, and representative pharmaceutical or biomedical applications.

Derivative and Modification	Key Feature/Application	Ref.
Thio-β-CD (high yield)	Mitsunobu reaction; easy thio-functionalization	[52]
Cup-shaped α-CD with aldehyde	Enhanced catalytic performance	[54]
β-CD–triazole hybrids	Rigid, water-soluble; increased prednisolone solubility; non-cytotoxic	[59]
Urea-substituted β-CD	Amphiphilic anion receptors; stable, water-soluble	[60]
Chitosan-functionalized β-CD	Anti-inflammatory activity	[61]
Mono-aldehyde & carboxyl β-CD derivatives	NaBH_4_/NaCNBH_3_ reductions; general route to tosyl-derived CDs	[62]
Solid-phase C-6 mono-substitution	Mild detachment on resin	[63,64,65]
Hollow CD nanospheres	Improved CPT stability; sustained release; high loading	[66,67]
β-CD blockers of anthrax toxin pores	Pore-blocking antivirulence agents	[68]
C_6_-aminated permethyl-CDs	Epoxide opening; aminoalcohol linkers; microwave-optimized yield	[69]
D-Carnosine-β-CD conjugates	Enzyme-resistant peptide delivery; enhanced stereoselective binding	[70]
Scutellarin-β-CD conjugates	Increased solubility, stability, cytotoxicity; antitumor activity	[71]
Maleic/itaconic acid–esterified β-CD	Phosphate-catalyzed esterification; 70/21% yields	[72]
Phospholipidyl-β-CD	Self-organizing amphiphiles; characterized by ESI-MS/MS	[73]
Trifluoromethylated β-CD	Artificial enzyme activity	[74]
Naphthalene-fluorophore β-CD	Fluorescent host–guest sensing; van der Waals–driven inclusion	[75,76]
Anthracene-β-CD	Good solubility; fluorescence profiling	[77]
Azobenzene-triazole-β-CD	Click-linked photoresponsive derivative	[78]
Primary-face modified CDs	Novel routes to C-6 functionalization	[79]

**Table 4:** Summary of preclinical studies on cyclodextrin-mediated gene delivery, including derivative type, genetic cargo, target cells, and key outcomes. Abbreviations: HP-β-CD = hydroxypropyl-β-cyclodextrin.

CD Derivative Used	Genetic Cargo	Target Cells/Tissue	Outcome/Key Findings	Ref.
HP-β-CD nanoparticle	siRNA targeting KRAS	Lung cancer cells	Enhanced cellular uptake, 60% knockdown efficiency; minimal toxicity	[142]
Methylated β-CD	mRNA encoding GFP	Hepatocytes	Increased transfection efficiency and protein expression in vitro and in vivo	[143]
Cationic Amphiphilic Cyclodextrin	mRNA for CFTR protein	Airway epithelial cells	Improved mRNA stability, efficient endosomal escape, restoration of CFTR function in cystic fibrosis model	[144]
Modified γ-CD nanoparticle	miRNA for oncogene silencing	Glioblastoma cells	Significant reduction in tumor growth and increased survival in animal models	[145]

**Table 5:** Clinical trials involving cyclodextrins and their derivatives, including disease indication, formulation, trial phase, and key outcomes.

Neurodegenerative Disease	CD Derivative	Target Lipid/Protein	Model/System Used	Observed Effects	Ref.
Alzheimer’s Disease	HP-β-CD	Cholesterol/Amyloid-β	Transgenic mouse model	Reduced amyloid deposition, improved memory	[150]
Parkinson’s Disease	HP-β-CD	α-Synuclein/Cholesterol	In vitro neuronal cultures	Decreased aggregation, enhanced neuronal survival	[150]
Huntington’s Disease	Methylated β-CD	Mutant huntingtin protein	Cellular models	Reduced aggregation, lowered cytotoxicity	[151]

**Table 6:** Applications of cyclodextrins in cancer therapy, summarizing CD-based systems, drug combinations, and therapeutic effects reported in preclinical models. ↑-increase; ↓-decrease.

Authors et al.	CD-Based System	Key Findings	Ref.
Dai et al.	Supramolecular nanoparticles: reduction-sensitive permethyl-β-CD–Camptothecin prodrug + adamantane–porphyrin photosensitizer + HA-TPP + β-CD	Mitochondrial uptake in A549 cells; in situ CPT release; ROS generation under light; synergistic chemo-photodynamic efficacy against lung cancer	[198]
Guimarães et al.	Cyclodextrin complexes of LGK974 (porcupine inhibitor)	↑ Solubility & bioavailability of LGK974; enabled safer, repeated oral/parenteral dosing; ↓ toxicity in Wnt-dependent tissues	[199]
Vaidya et al.	Β-CD–erlotinib inclusion coated with PLGA	Enhanced NSCLC cell uptake; lowered IC_50_; suppressed colony formation; ↑ apoptosis; inhibition of autophagy	[200]
Wang et al.	Sulfobutylether-β-CD–resveratrol complexes loaded on polymeric nanoparticles	↑ Cellular uptake, cytotoxicity & apoptosis in NSCLC models; maintained resveratrol’s antioxidant activity; superior efficacy vs. Free drug	[201]
Shukla et al.	CD-derivative–Celastrol complex	Improved intestinal permeability & physiological stability; enhanced cytotoxicity in human lung cancer cells	[202]
Lin et al.	Β-CD–polycaprolactone block copolymer conjugated with folic acid—metformin carrier	pH-responsive metformin release (faster at pH 6.4); folate-receptor-mediated uptake in A549; controlled release and targeted anti-tumor efficacy with low toxicity	[203]

**Table 7:** Overview of cyclodextrin-based strategies in anticancer therapy, summarizing cancer type, formulation approach, and therapeutic benefits.

Authors	Cancer Type	CD-Based System	Key Findings	Ref.
Hyun et al.	Breast cancer	β-CD + polyethylene glycol + folic acid nanocarrier loaded with doxorubicin (IV administration in animals)	Decreased tumor volume after IV dosing; no systemic toxicity or cardiotoxicity—targeted, safer DOX delivery	[204]
Farrokhi et al.	Breast cancer	β-CD polymer nanocarrier delivering an RNA-cleaving DNAzyme targeting c-Myc (tested in MCF-7 cells)	Synergistic inhibition of MCF-7 proliferation when combined with doxorubicin—enhanced anticancer activity	[205]
Mihanfar et al.	Breast cancer	β-CD-functionalized dendrimeric graphene-oxide magnetic nanoparticles loaded with doxorubicin	Increased proliferation inhibition and apoptosis; reduced off-target DOX side effects in vivo; GO may sensitize cancer cells	[206]
Zafar et al.	Breast cancer	β-CD complexes of genistein with D-α-Tocopherol PEG1000 succinate (TPGS)	Improved genistein solubility → significantly greater antioxidant and cytotoxic activities vs. free genistein	[207]
Lee et al.	Breast cancer	β-CD + polyethylene glycol + folic acid carrier for adamantane–NIRF conjugate	Highly efficient tumor targeting and excellent breast-tumor targetability	[208]
Panagiotakis et al.	Breast cancer	Permethyl-β-CD complexes with water-insoluble photosensitizers (meso-tetraphenylporphyrin & analog)	Photostability, strong intracellular fluorescence, high photokilling efficiency and low dark toxicity in MCF-7 cells	[209]
Soleimani et al.	Breast cancer	Magnetic nanogel: β-CD + poly(2-ethyl-2-oxazoline) + iron-oxide NPs, loaded with doxorubicin HCl	High drug loading, slow/stimuli-triggered release, good cytocompatibility; enables combined chemotherapy + hyperthermia	[210]
Ercan et al.	Breast cancer	Blank 6-O-Caproyl-β-CD nanoparticles (administered to MCF-7 cells)	Increased levels of apoptosis-related proteins and prevention of cell proliferation (intrinsic apoptotic effect)	[211]
Kasinathan et al.	Breast cancer	Hybrid nanocomposite of β-CD and molybdenum disulfide (MoS_2_)	Potent inhibition of MCF-7 cells plus antibacterial properties—promising for cancer therapy	[212]
Baskar & Supria Sree	Prostate cancer	β-CD–chitosan nanobiocomposite loaded with l-asparaginase	Good anticancer activity vs. prostate cell lines; IC_50_ ≈ 125 µg/mL (≈ half the concentration of free enzyme)	[213]
Trindade et al.	Prostate cancer	Inclusion complexes of β-CD with carvacrol	Dose-dependent inhibition of tumor cells in 2D/3D cultures; potent antiproliferative effects against PC-3 cells	[214]
Kost et al.	Cervical cancer	β-CD core complexed with doxorubicin, loaded into stereocomplexed polylactide micelles (SCMs)	Controlled DOX release and more efficient tumor cell suppression vs. free drug	[215]
Reis et al.	Cervical cancer	Gold-core/silica-shell (AuMSS) NPs coated with poly-2-ethyl-2-oxazoline (PEOZ) and β-CD (various ratios)	Improved biological performance, enhanced cytocompatibility and increased internalization in HeLa cells	[216]
Russo Spena et al.	Ovarian cancer	Modified CD encapsulating a Pin1 inhibitor, remotely loaded into PEGylated liposomes	Preferential tumor accumulation, favorable PK; induced proteasome-dependent degradation of Pin1—promising antitumor effects	[217]

## Data Availability

No new data were created or analyzed in this study. Data sharing is not applicable.
